# Characterizing Electrogram Signal Fidelity and the Effects of Signal Contamination on Mapping Human Persistent Atrial Fibrillation

**DOI:** 10.3389/fphys.2018.01232

**Published:** 2018-09-05

**Authors:** David Vidmar, Mahmood I. Alhusseini, Sanjiv M. Narayan, Wouter-Jan Rappel

**Affiliations:** ^1^Department of Physics, University of California, San Diego, San Diego, CA, United States; ^2^Division of Cardiology, Department of Medicine, Stanford University, Stanford, CA, United States

**Keywords:** atrial fibrillation, phase map, signal fidelity, signal contamination, modeling

## Abstract

**Objective:** Determining accurate intracardiac maps of atrial fibrillation (AF) in humans can be difficult, owing primarily to various sources of contamination in electrogram signals. The goal of this study is to develop a measure for signal fidelity and to develop methods to quantify robustness of observed rotational activity in phase maps subject to signal contamination.

**Methods:** We identified rotational activity in phase maps of human persistent AF using the Hilbert transform of sinusoidally recomposed signals, where localized ablation at rotational sites terminated fibrillation. A novel measure of signal fidelity was developed to quantify signal quality. Contamination is then introduced to the underlying electrograms by removing signals at random, adding noise to computations of cycle length, and adding realistic far-field signals. Mean tip number *N* and tip density δ, defined as the proportion of time a region contains a tip, at the termination site are computed to compare the effects of contamination.

**Results:** Domains of low signal fidelity correspond to the location of rotational cores. Removing signals and altering cycle length accounted for minor changes in tip density, while targeted removal of low fidelity electrograms can result in a significant increase in tip density and stability. Far-field contamination was found to obscure rotation at the termination site.

**Conclusion:** Rotational activity in clinical AF can produce domains of low fidelity electrogram recordings at rotational cores. Observed rotational patterns in phase maps appear most sensitive to far-field activation. These results may inform novel methods to map AF in humans which can be tested directly in patients at electrophysiological study and ablation.

## Introduction

Fibrillation is the most common form of cardiac arrhythmia. Ventricular fibrillation (VF) is life-threatening and responsible for over 300,000 cases of sudden cardiac arrest per year (Nichol et al., 2008; Tracy et al., 2012), while atrial fibrillation (AF) affects 30 million people worldwide and is a major cause of stroke and debility (Chugh et al., 2014; January et al., 2014). Despite decades of research, the precise mechanisms underlying fibrillation are still debated (Pandit and Jalife, 2013; Nattel et al., 2017). Progress is slow in large part due to the inherent difficulty in mapping complex rhythms in humans where optical mapping, commonly used in animal studies (Gray et al., 1995; Mandapati et al., 2000), is not feasible. Therefore, the most accurate clinical determination of activation patterns of human AF and VF must come from the use of high density electrode arrays (Narayan et al., 2012a; Krummen et al., 2014) or non-invasive body surface mappings (Ramanathan et al., 2004).

Recent studies using intracardiac basket electrodes have revealed that during VF and AF spiral waves, or rotors, may underlie the irregular tissue activity (Narayan et al., 2012a,b; Krummen et al., 2015), recently confirmed by other intracardiac methods (Daoud et al., 2017; Grace et al., 2017) and with non-invasive mapping techniques (Haissaguerre et al., 2014). The relevance of these spiral waves have been supported by computational studies (Rappel et al., 2015) and by targeted ablation (Narayan et al., 2012c) and may more rapidly result in AF termination than traditional ablation to electrically isolate the pulmonary veins (Haissaguerre et al., 2014). Furthermore, the 1-year success of such ablation techniques is higher than the 40–60% reported for pulmonary vein ablation alone in many studies (Haissaguerre et al., 2014; Sommer et al., 2016; Miller et al., 2017; Spitzer et al., 2017) although meta-analyses show variable outcomes between centers (Ramirez et al., 2017; Baykaner et al., 2018; Parameswaran et al., 2018).

Nevertheless, these data are debated since <50% of patients show acute termination, and some groups report difficulties obtaining good clinical results with heterogeneity in meta-analyses (Ramirez et al., 2017; Baykaner et al., 2018; Parameswaran et al., 2018). One possible explanation for discrepant results, particularly in patients who have failed multiple prior procedures, might be variations in signal quality with subsequent degradation of mapping accuracy. In this study, we propose a method for quantifying signal fidelity and systematically determine how signal contamination affects stable rotational activation during clinical AF using an intracardiac electrode array. To show the potential of the fidelity metric and the possible effects of signal contamination, we focus on data obtained from several patients with AF. In future work we plan to apply these results to improve mapping and ablation in large patient cohorts.

## Methods

### Data processing

We will focus here on signals recorded from 64-pole basket electrodes on an 8 × 8 grid, recorded clinically during AF at a sampling rate of 977 Hz upsampled to 1,000 Hz. These data were recorded in the left atrium of patients with persistent AF in whom anti-arrhythmic medications had been withheld for 72 h prior to ablation. During the procedure, carried out at the Stanford University Hospital, Palo Alto, CA, ablation acutely terminated persistent AF to sinus rhythm. The study was approved by the IRB.

Quality of electrogram signals in AF was determined using a signal fidelity measure developed below, from which the majority of electrograms signals were of high quality with few contaminants. Electrograms spanning a time interval of 20 s, corresponding to over 400 cycles of AF, are analyzed. QRS contamination is removed from each signal by subtracting the average QRS complex from the electrode signal at each R wave_._ All traces are filtered with a 1.5–25 Hz Butterworth bandpass filter.

In order to allow for computation of phase maps from these signals we use a technique, recently proposed by Kuklik et al. (Kuklik et al., 2015, 2016), of sinusoidally recomposing the signals before applying the Hilbert Transform. In contrast to some other techniques, this technique is published and publicly available (http://narayanlab.stanford.edu). This technique has been compared to distinct mapping methods, and shows rotational activity in AF at similar sites, including sites of termination of persistent AF by ablation (Alhusseini et al., 2017). Briefly, the dominant cycle length of each electrogram is computed as that with largest power between 130 and 280 ms in the Welch spectral density estimate of the signal (window size = 2,000 ms, overlap = 1,000 ms). A recomposed signal is then computed as a sum of single-period sinusoidal waves, with period corresponding to the dominant cycle length and with amplitude corresponding to the deflection amplitude of the signal (–dV/dt, with positive deflections set to zero). To obtain spatial maps of the activation patterns, we apply the Hilbert Transform (Gray et al., 1998; Bray et al., 2001; Umapathy et al., 2010) to compute complex analytic signals which are interpolated in space and used to determine instantaneous phase. All spatial maps in this study are interpolated using linear interpolation to a grid with 225 × 225 points.

### Signal fidelity

**Figure 1:**
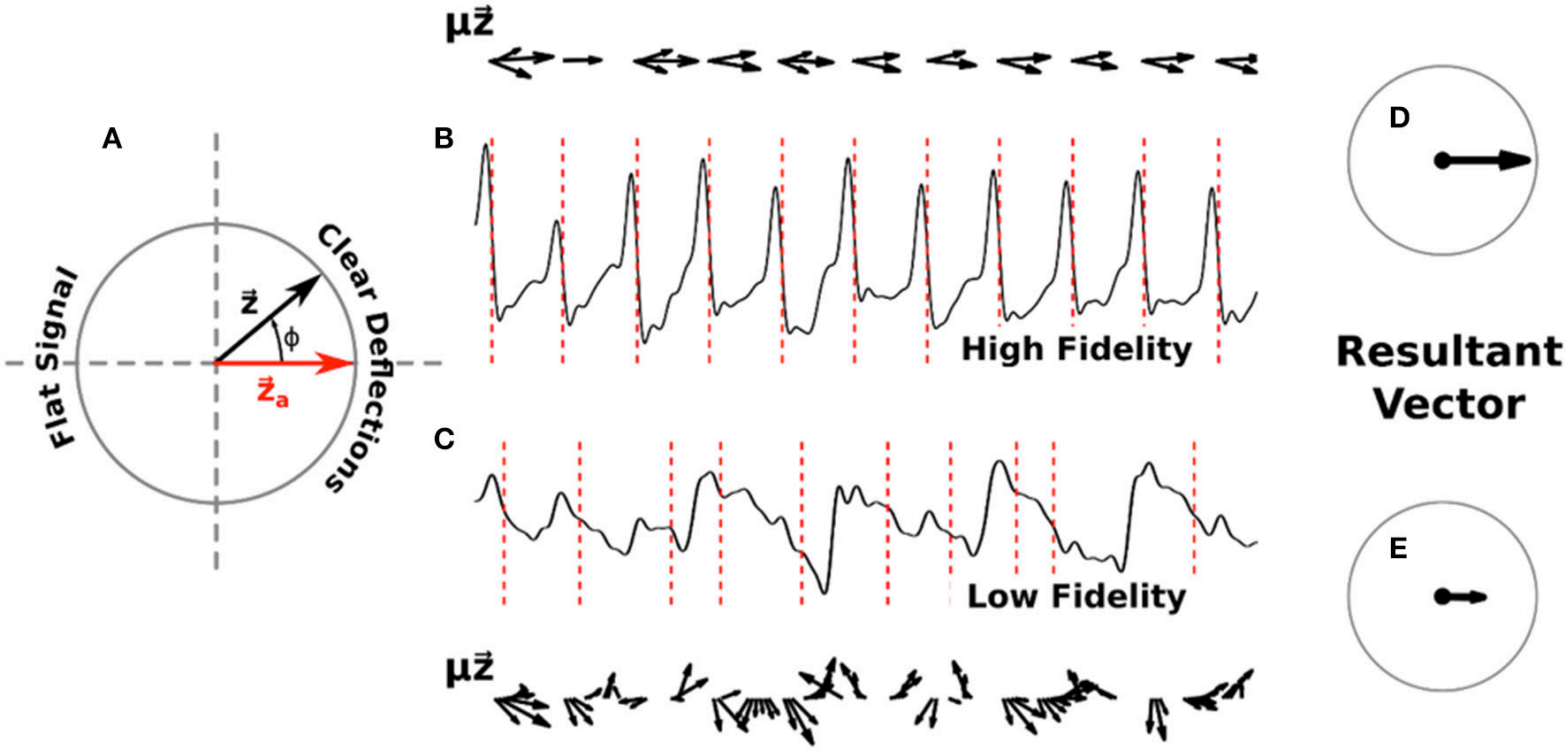
Diagram of signal fidelity computation. **(A)** A visual representation of phase in the complex plane. The red vector ${\overset{⇀}{z}}_{a}$ represents activation, with the complex phase vector $\overset{⇀}{z}$ rotating counter-clockwise around the unit circle as determined by the computed phase of a given signal. **(B)** A sample filtered electrogram with high signal fidelity, exhibiting clear deflections which are well-marked by the computed activations (red dotted lines). Phase vectors $\overset{⇀}{z}\left(t\right)$, scaled by the deflection amplitude μ(*t*), are shown above this trace in 10 ms increments. **(C)** A sample filtered electrogram with low signal fidelity, exhibiting ambiguous deflections. Phase vectors $\overset{⇀}{z}\left(t\right)$, scaled by the deflection amplitude μ(*t*), are shown below this trace in 10 ms increments. For clarity, vectors with magnitude below a minimum threshold are not shown. Panels **(D)** and **(E)** show the normalized vectors resulting from the sum of scaled phase vectors for each respective trace across 20 s of data. Signal fidelity is computed as the dot product of this resultant vector and ${\overset{⇀}{z}}_{a}$.

We defined a measure of signal fidelity to determine the faithfulness of phase maps to underlying electrograms. Let us represent the computed phase ϕ(*t*) of a given signal as a complex phase vector $\overset{⇀}{z}\left(t\right)\;=\;e^{i\phi \left(t\right)}$ with each activation occurring as this vector passes the positive real axis, denoted as ${\overset{⇀}{z}}_{a}$. This unit vector can be visualized as a counterclockwise rotating vector, as depicted in Figure 1A.

To define a time-averaged signal fidelity, Γ, for each electrogram we note that signals which exhibit high fidelity should have distinct negative deflections, i.e., large –dV/dt, at the times when $\overset{⇀}{z}$ is near an activation ${\overset{⇀}{z}}_{a}$, and should have minimal deflections otherwise. Conversely, signals with low fidelity might exhibit ambiguous deflections occurring when $\overset{⇀}{z}$ is not near ${\overset{⇀}{z}}_{a}$, or could have small negative deflections around ${\overset{⇀}{z}}_{a}$. Thus, we can define

(1) $$\Gamma =\;\left[\frac{\sum_{t}\mu \left(t\right)\vec{z}\left(t\right)}{\sum_{t}\mu \left(t\right)}\right]\;\cdot {\vec{z}}_{a}\;=\;\frac{\sum_{t}\mu \left(t\right)\cos\phi (t)}{\sum_{t}\mu \left(t\right)}$$

where μ is equal to the magnitude of the derivative of the signal if it is negative (i.e., μ = |–dV/dt|) and zero otherwise, and where the sum is over the entire time interval. This fidelity ranges from −1 to +1, with larger values corresponding to easily interpretable and high fidelity signals and smaller values indicating low fidelity signals with relatively indistinct deflections.

In Figures 1B,C we show example computations of the signal fidelity measure across multiple deflections for two sample electrograms, one with high fidelity (Γ = 0.90) and one with low fidelity (Γ = 0.43). These electrograms were taken from a 67 years old female patient with a left atrial size of 55 mm and a left ventricular ejection fraction of 36%. On the top/bottom of these signals we show the weighted vectors $\mu \overset{⇀}{z}$ from Equation (1) in increments of 10 ms. Summing up all of these vectors for the entire trace and normalizing gives us the resultant vectors shown in Figures 1D,E. Signal fidelity is then the dot product of this resultant vector and ${\overset{⇀}{z}}_{a}$.

All of the vectors $\overset{⇀}{z}$ in the high fidelity trace occurring at or near activations also exhibit large deflection*s μ*, with vectors $\overset{⇀}{z}$ occurring away from activations exhibiting small or non-negative deflections. The sum in Equation (1), therefore, is taken over coherent vectors pointing mostly along the real axis resulting in a high fidelity measure. In the low fidelity trace, however, many vectors $\overset{⇀}{z}$ occurring at phases different from activation also exhibit large deflections. The origin of these large deflections at times different from activation are currently not clear but may include far-field effects, structural heterogeneities, and motion artifacts. The sum of these incoherent vectors, which can vary in direction as well as amplitude, results in a smaller resultant vector indicating a lower fidelity measure.

### Tip density

To quantify rotational dynamics we compute phase singularities (PS) on our phase maps using a standard approach (Gray et al., 1998; Bray et al., 2001; Umapathy et al., 2010), corresponding to the location of spiral wave tips, and determine the mean total number of tips *N*. Further, we can define phase singularity maps which quantify the amount of clockwise, Ω_CW_ (*t*), and counter-clockwise rotation, Ω_CCW_ (*t*), such that Ω = 1 if there is a phase singularity of the given chirality within a distance of 28 interpolated grid points of each location, and Ω = 0 otherwise. Note that this distance is slightly smaller than the spacing of one electrode. Because we are primarily interested in stable, rather than transient, rotational patterns, we compute the density of spiral tips over time as

(2) $$\delta \;\overset{\text{def}}{=}\frac{1}{T}\sum_{t}\left[\Omega_{CW}\left(t\right)\;-\;\Omega_{CCW}(t)\right],$$

where *T* is the total duration of the signal. This density ranges from −1 to +1, with larger positive (negative) values indicating a region of tissue which experienced consistent clockwise (counter-clockwise) rotation for a significant portion of the episode. In addition, for each chirality we can then use k-means clustering, via MATLAB function k-means, to determine the largest spatial cluster of PS locations.

### Signal contamination

In this study, we investigated three potential sources of signal contamination in AF recordings: (1) Non-viable data due to signal saturation, (2) reduced signal quality due to poor electrode contact, and (3) false deflections from far-field activity. We quantified the effects of these sources on the mean tip number and tip density using a large number of random trials, further specified below.

First, we randomly removed signals from our recordings to simulate saturated data. This is motivated by clinical observations of electrogram data in which revealed that >5–10% of electrode sides may exhibit poor quality electrograms. Possible causes for loss of quality include poor size match of the basket with intermittent contact [which we circumvent clinically using matched basket sizes and multiple positions (Zaman et al., 2017)], electrical interference such as contact with an ablation electrode causing saturation, or other factors.

Along each affected spline, linear interpolation/extrapolation of recomposed signals was used to account for electrodes with no data and we quantify the corresponding effect as a function of the number of removed signals. Second, to determine the effects of incorrect cycle length determination due to poor signal quality we add independent normally distributed noise with standard deviation σ to the computed dominant cycle lengths of each electrogram. Third, we choose a single region of raw electrogram signal exhibiting a clear deflection to use as a surrogate deflection for far-field activity. Such far-field activity might arise from structural heterogeneities, including differences in wall thickness. This surrogate deflection is added at random times to the raw electrograms, being sure not to overlap surrogates. The amplitude of these deflections is set to some constant multiplied with the standard deviation of the filtered signal. Both the constant and number of deflections are varied.

## Results

### Signal fidelity

**Figure 2:**
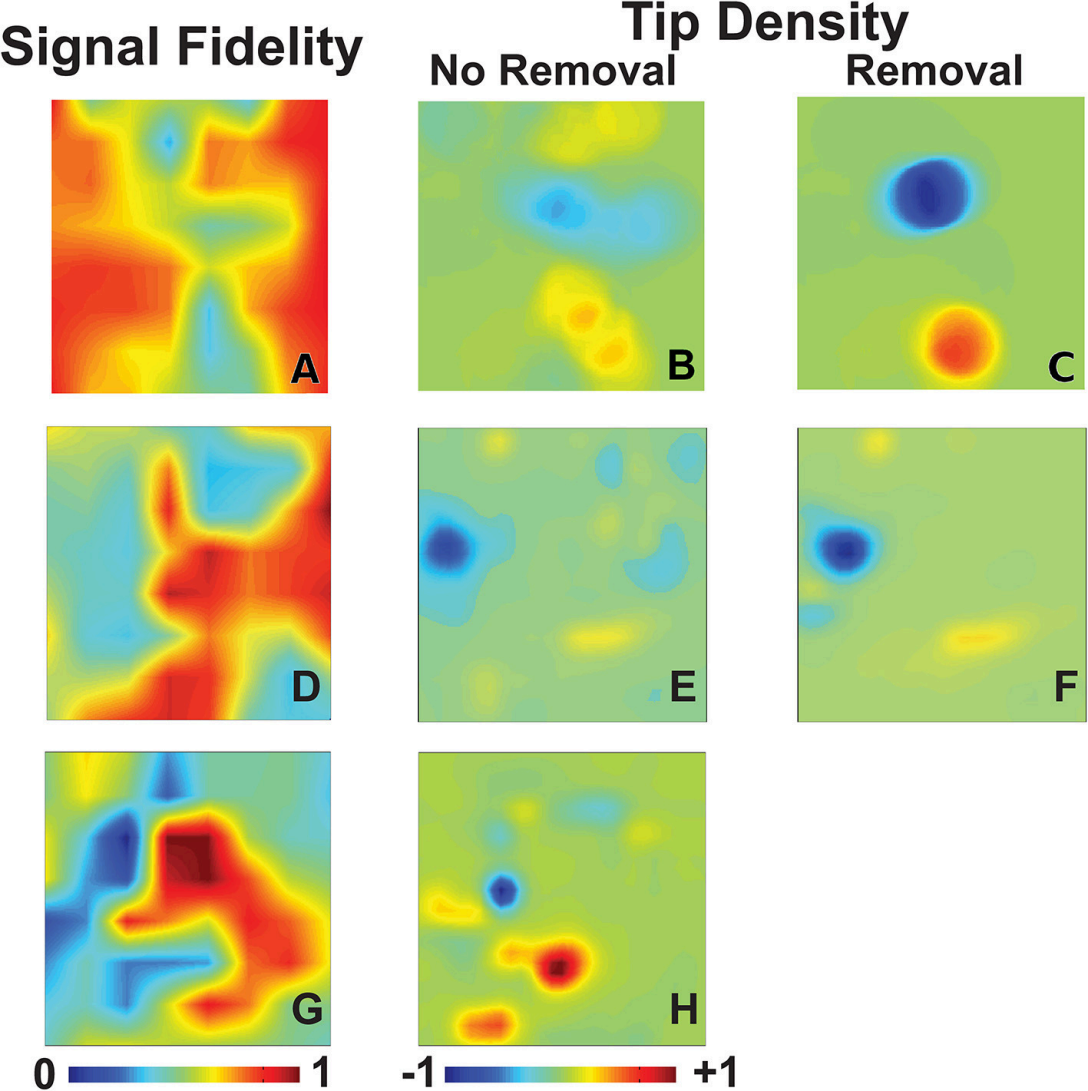
Signal fidelity in patients during AF. **(A–C)** Signal fidelity map **(A)** for an episode of human AF corresponding to the electrograms shown in Figure 1 (67 years-old female patient). The corresponding tip density map, with clockwise rotations in red and counter-clockwise rotations in blue, and tip density map after k-means removal, and interpolation over, electrogram recordings with low fidelity are shown in **(B)** and **(C)**, respectively. High values of tip density show concordance with domains of low fidelity. The magnitude of maximum tip density around the termination site (blue peak) was 0.43. The magnitude of maximum tip density around the termination site increases significantly to 0.94. **(D–F)** Signal fidelity map **(D)**, tip density map **(E)**, and tip density map after removal of low fidelity electrodes **(F)** corresponding to an episode of human AF of a 66 years old male patient. **(G–H)** Signal fidelity map for an episode of human AF of a 49 years old male patient **(G)** and the corresponding tip density map **(H)**.

We first examine the signal fidelity map of AF for the patient whose electrograms are shown in Figure 1. This map shows two distinct domains of fidelity values, with a majority of the tissue exhibiting large values of fidelity and a minority exhibiting low values of fidelity (Figure 2A). The corresponding tip density map for this episode is shown in Figure 2B, where regions of elevated positive or negative values of density correlate well with visually-observed rotational activity in animations of phase maps (see Supplementary Video). Particularly, the region of peak CCW rotation in these tip density maps, shown in dark blue, corresponds to the site where ablation terminated AF in this patient. The peak magnitude of CW and CCW tip densities are 0.36 and 0.43, respectively. Due to the spatial meander of the computed phase singularities, as reported in our earlier clinical reports (Narayan et al., 2012a, 2013) and in animal studies (Zlochiver et al., 2008), the tip density map shows spatially extended regions for both CW and CCW rotational activity. Interestingly, the region of elevated tip density also coincided with low values of fidelity.

With our method of computing signal fidelity we can quantify the expected faithfulness of signals to the underlying conduction. Signals which have low fidelity, then, can be discarded and interpolated over if desired. A convenient method of partitioning our signals into groups of either high or low fidelity uses k-means clustering, an iterative algorithm which clusters observations into *N* groups with similar values (Hartigan, 1975). We demonstrate this in Figure 2C, where we use k-means clustering on all values of signal fidelity to partition each signal into one of two groups. This separates our recordings into a domain of signals with high fidelity (*n* = 44) and those with low fidelity (*n* = 20). The signals marked by k-means as belonging to the low fidelity group are discarded. As outlined in Methods, discarded signals are accounted for through interpolation/extrapolation of recomposed signals along each spline. This removal of poor quality signals, in this case potentially due to fractionation expected at the core of rotational activity (Nademanee et al., 2004), results in a significant increase in the magnitudes of CW and CCW tip densities to 0.61 and 0.94, respectively.

Two additional patients in whom ablation acutely terminated persistent AF to sinus rhythm are presented in Figures 2D–H. One patient, a 66 years old gentleman, had a left atrial size of 47 mm and a left ventricular ejection fraction of 59%. Computing phase maps and phase singularities revealed a region of elevated CCW tip density as shown by the blue area in the Figure 2D. The location of this CCW rotor coincided with the ablation target which converted AF to sinus rhythm. As in the patient of Figures 2A–C, this region also coincided with low values of fidelity (Figure 2E) and removing electrodes with small values of fidelity (in this case the *n* = 16 electrodes for which Γ < 0.5) increased the tip density (Figure 2F). In this case, however, the increase was modest (approximately 10%).

The other additional patient was a 49 years old gentleman with a left atrial size of 53 mm and a left ventricular ejection fraction of 51%. The phase density maps reveal a more complex pattern with a clear CCW and several CW rotors (Figure 2G). Again, the location of these elevated regions of rotation correlate well with regions of low fidelity (Figure 2H). Furthermore, the location of the CW rotation (red) corresponds with the site of ablation that terminated AF to sinus rhythm. Likely due to the complexity of the pattern, removal of electrodes was not successful in increasing the phase singularity density.

### Effect of non-viable signals

**Figure 3:**
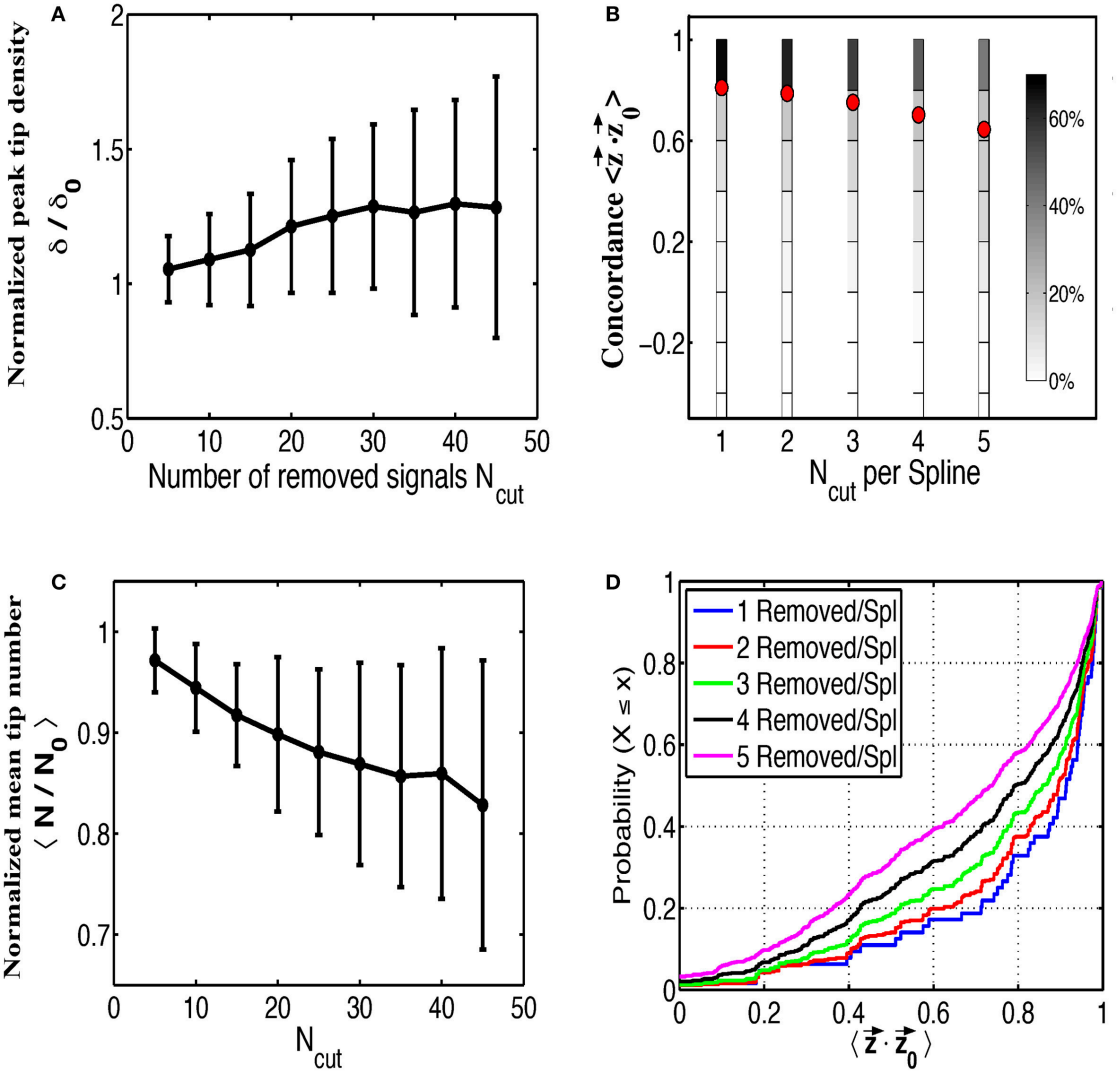
The effects of random removal of electrodes. Normalized plots of peak tip density around the site of termination **(A)**, and mean number of tips **(B)**, as a function of the number of electrodes removed are shown for 200 trials. Error bars show standard deviation. Electrodes are then systematically removed per spline and the concordance between the interpolated phase vectors $\overset{⇀}{z}$ and the original phase vectors ${\overset{⇀}{z}}_{0}$ is shown as multiple histograms in **(C)**, with red dots marking the mean, and as cumulative distribution functions in **(D)**.

Next we examined the impact of various sources of contamination on the ability to identify rotational activity in phase maps, taking the patient corresponding to Figures 2A–C as an example. We first removed signals at random from our grid, leaving at least two electrodes on any spline since complete loss of a spline is rare, to simulate saturation rendering some signals non-interpretable. In Figure 3A we plot the peak tip density δ around the termination site, as well as the mean tip number *N* in Figure 3B, as a function of the number of removed signals. Both of these plots are normalized to their respective values when no electrodes have been removed and are computed as the mean result over 200 random trials. Figure 3A shows that as more and more electrodes are randomly removed, the magnitude of tip density first increases, then reaches a plateau. In Figure 3B, mean tip number, however, continually decreases as we increase the number of removed electrodes across our entire range. Note that for both figures the standard deviation equals 0 for *N*_cut_ = 0 and becomes larger when the number of randomly removed electrodes is increased.

In addition to determining the effect on the phase maps, we also examined the accuracy of interpolating over removed signals. To do this we removed between 1 and 5 signals along each spline and interpolate across these signals. This was repeated for each permutation of removed electrodes along each spline. We then compared the resulting phase from this interpolated signal ϕ (*t*) to the phase from the original removed signal ϕ_0_ (*t*) to determine how well our interpolated phase matches with the original phase.

To quantify concordance we computed time-averaged dot product of the interpolated phase vector $\overset{⇀}{z}$ with the original phase vector ${\overset{⇀}{z}}_{0}$. This dot product ranges from −1 to 1, with large positive values implying very similar phase progression and negative values corresponding to an interpolated phase that is, on average, out of phase with the original phase. The results of this procedure over all permutations and for all number of removed electrodes, are shown in Figure 3C as multiple histograms with red dots denoting the average. This plot demonstrates that interpolation shows relatively high concordance with the missing data, particularly if fewer electrodes are missing per spline. With increasing number of removed electrodes per spline, the distribution of concordance values becomes less peaked near unity implying a greater chance that interpolated phase poorly represents the underlying missing data. Cumulative distribution functions for each number of removed electrodes per spline are shown in Figure 3D for a more precise comparison of the concordance distributions.

### Effect of varying cycle length

**Figure 4:**
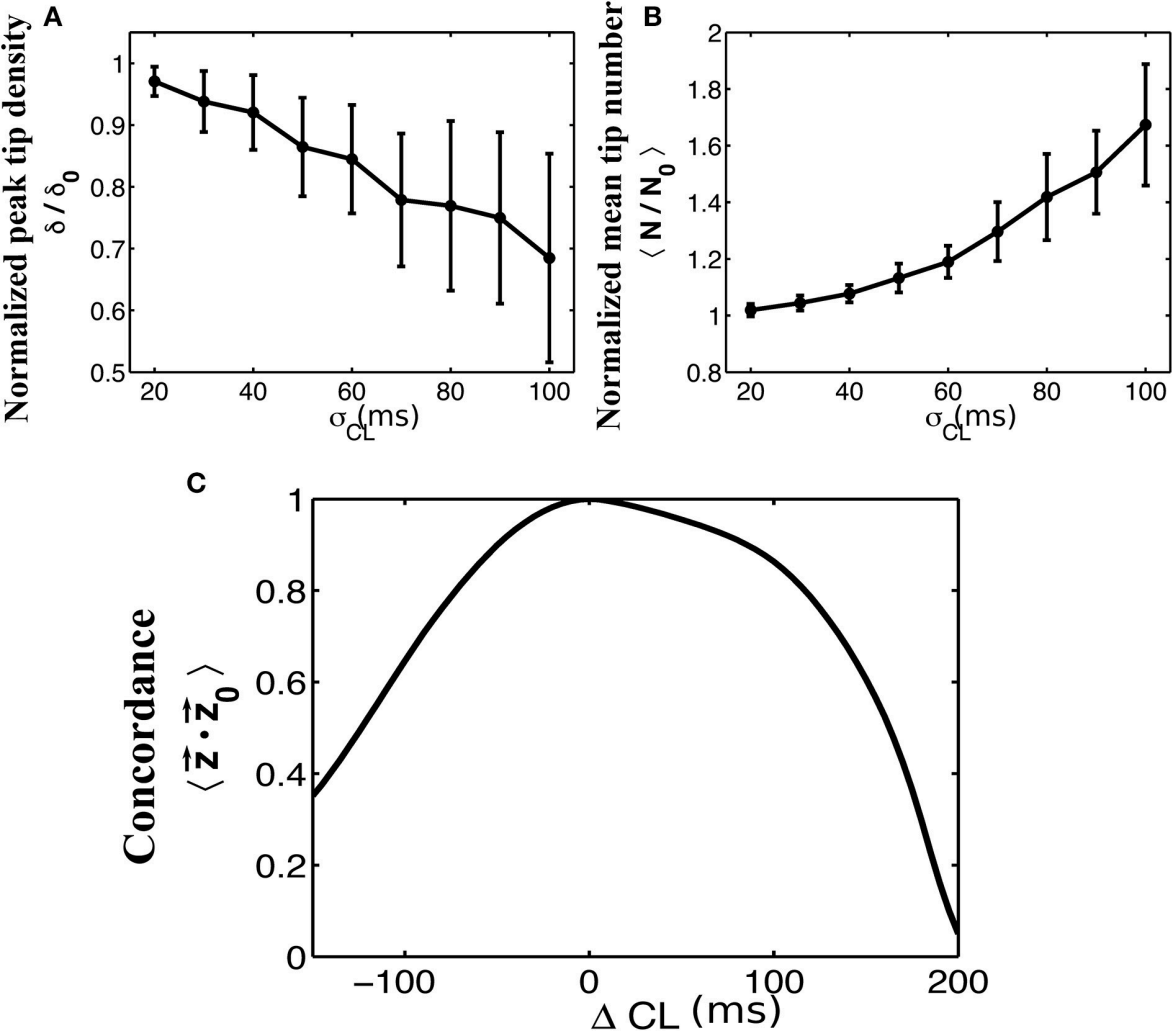
The effects of adding normally distributed noise to the dominant cycle length. Normalized plots of peak tip density around the site of termination **(A)**, and mean number of tips **(B)**, as a function of the standard deviation of noise added to the dominant cycle lengths are shown for 50 trials. Error bars show standard deviation. A minimum cycle length of 50 ms is used throughout all trials. **(C)** Mean concordance between the resulting phase vectors $\overset{⇀}{z}$ and the original phase vectors ${\overset{⇀}{z}}_{0}$ after systematically adding values between −150 and 200 to the originally determined cycle lengths.

A critical part in the sinusoidal reconstruction of our underlying signal is the estimation of the dominant cycle length. To examine the possibility of poor quality recordings resulting in inaccurate cycle lengths, we add a noise term to each computed value of dominant cycle length before reconstructing the signals. We set a minimum cycle length of 50 ms to ensure that this never results in a negative cycle length. The results of this process as a function of the standard deviation σ of our normally-distributed noise term are shown in Figure 4. As the standard deviation of our noise term increased, tip density at the termination site decreased and the total number of tips increases. This effect was further examined in Figure 4C, where we systematically added values from −150 to 200 to each computed dominant cycle length. Again, we measured the resulting average concordance between phase vectors $\overset{⇀}{z}\;$resulting from these altered cycle lengths and the original phase vectors ${\overset{⇀}{z}}_{0}$.

### Effect of far-field contamination

**Figure 5:**
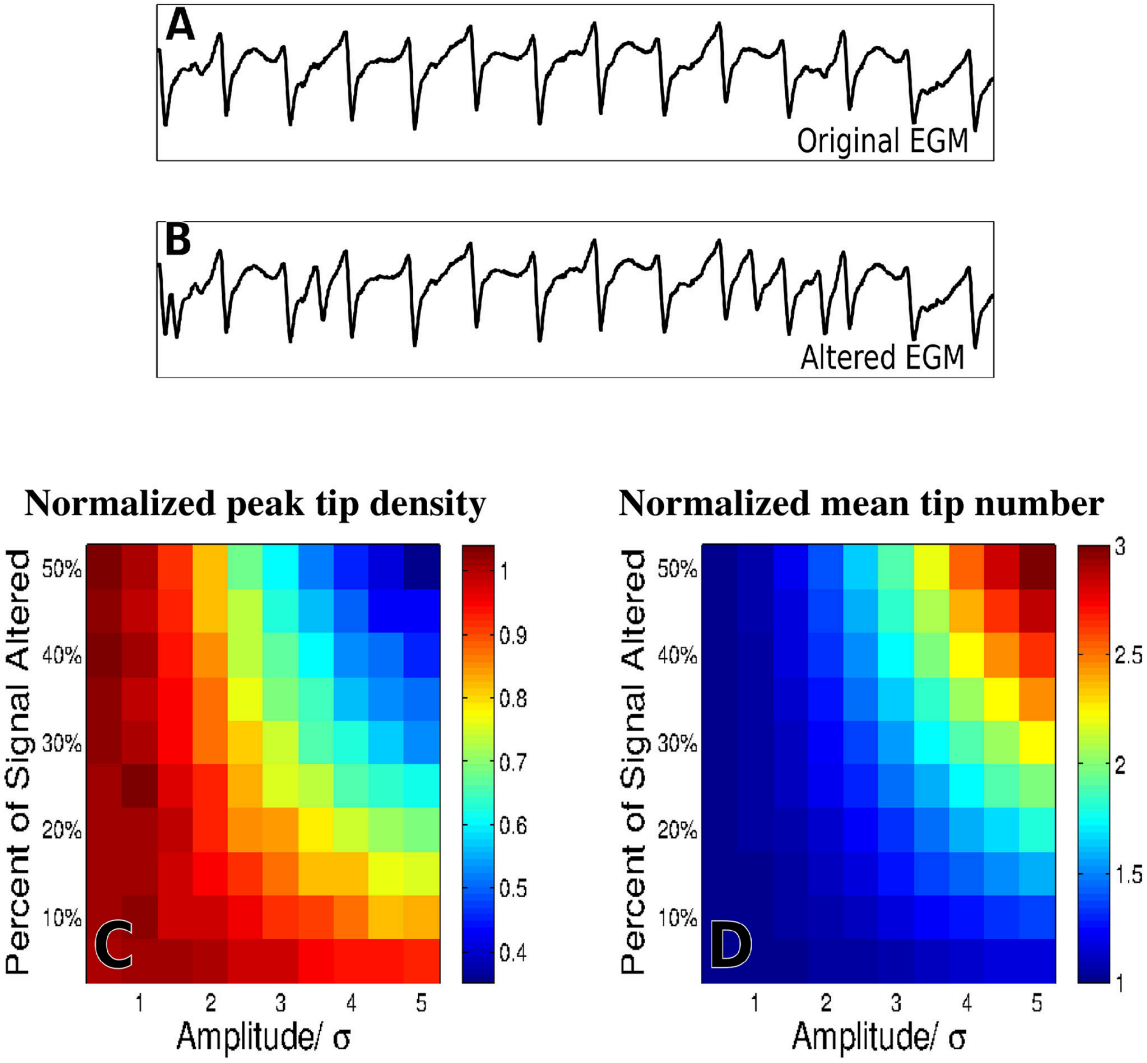
The effects of adding false deflections to electrograms. A sample EGM before adding false deflections **(A)** and after altering 30% of the signal with deflections of amplitude/σ = 3 **(B)** Normalized heat maps of peak tip density around the site of termination **(C)**, and mean number of tips **(D)**, as a function of both amplitude and frequency of added deflections are shown for 50 trials.

To evaluate the effects of far-field contamination we add surrogate deflections at random to the raw electrograms, as is shown in Figures 5A,B, and compute phase maps. The resulting tip density and tip number are shown as heatmaps in Figure 5 for different deflection amplitudes (0.5–5.0 times the standard deviation of the filtered signal, increments of 0.5) and different percentages of altered signal (5–50% signal altered, increments of 5%). Figure 5C shows that for large values of both frequency and amplitude of far field deflections, the tip density at the termination site decreases to less than half its original value. Similarly, Figure 5D shows that in this same domain the mean tip number increases by a factor of three, implying that far-field deflections can cause an increase in false detection of transient rotational activity.

## Discussion

In this study we set out to quantify the impact of variations in signal fidelity and the effects of electrogram contamination on mapping human AF to identify potential rotational drivers. Using recordings from patients in whom ablation at a site of stable rotational activity terminated persistent AF, we generated phase maps using a validated, non-proprietary method. Systematic addition of contamination and other perturbations revealed two main findings. First, sites of stable rotational activity in the animated phase maps corresponded to regions of low signal fidelity. Second, we found that observed rotational activity in AF was relatively robust to both removing signals at random and to altering dominant cycle lengths, whereas it was sensitive to the addition of surrogate far-field deflections. These findings have implications for future improvements in AF mapping, which may help to improve ablation targeting and patient outcomes.

### Signal fidelity at rotor core sites

The concept that electrograms at rotational cores should exhibit unique characteristics has been proposed in many different forms, whether through complex fractionation (CFAE) (Nademanee et al., 2004), larger values of Shannon entropy in bipolar electrograms (Ganesan et al., 2013), or higher dominant frequency (Zlochiver et al., 2008). While recent studies reported difficulty in finding distinguishing characteristics in electrograms at sites of rotational activity (Narayan et al., 2013; Benharash et al., 2015), this study shows that such sites may be identified by our proposed metric of electrogram signal fidelity. Our measure of signal fidelity, which quantifies how distinct the deflections of an electrogram are from their baseline, may be able to identify spiral wave cores. Although the precise reason for this observed lower fidelity is not clear, we postulate that increased tip meander and reduced electrogram amplitudes may contribute to more irregular electrograms. Studies are ongoing to validate this finding and to pinpoint the responsible mechanism in larger series in our Institutions.

The developed measure of signal fidelity may enable the targeted removal of signals which are difficult to mark, using information from high fidelity neighboring electrodes to infer their value. As a result of the removal of the electrodes near the spiral tip, electrodes that capture the spiral arms define the activation pattern, potentially improving spiral wave identification. Using a clustering algorithm, such as k-means, or removing electrodes with a lower fidelity than a certain threshold, this can be automated. In this study, the k-means removal of low fidelity recordings significantly increased peak tip density while the removal of 25% of electrodes, corresponding to those with the lowest fidelity, resulted in a modest increase in peak tip density. These possibly surprising findings are important because it implies that the original regions of elevated tip density were not a result of the low fidelity recordings (implying false positive tip detection), but rather that elevated tip density may have been a direct result of large-domain rotational circuits surrounding low fidelity regions. Thus, the low signal fidelity observed may be a result of rotational activity, as suggested theoretically (Pandit and Jalife, 2013) and consistent with recent reports which estimated the required spatial resolution for rotational activity (Rappel and Narayan, 2013).

### Insensitivity of mapping rotational activity to signal “dropout”

Observed rotational activity in this study was relatively robust to both removing signals at random and to altering dominant cycle lengths, yet sensitive to the addition of surrogate far-field deflections. These results are summarized in Figures 3–5, where we ran multiple trials adding in these contaminants at random for the patient corresponding to Figures 2A–C. Of note, by removing signals at random and interpolating over the missing signals the tip density at the termination site actually increases. This is true even when removing over half of the signals present. Again, this suggests that observed rotational activity is not due to a small handful of signals but is rather an emergent behavior of the system as a whole. Removing any given electrode did not appear to have a significant impact, and in fact removing certain low-fidelity electrodes, as previously discussed, improved the detection of rotational activity. Whether this finding is generalizable will likely depend on the spatial domain of rotational circuits, because small domain rotations spanning only a handful of electrodes may be easily obscured by electrogram removal. Moreover, as more electrodes are removed, their interpolation becomes less reliable as is shown in Figures 3C,D.

### Far-field contamination degraded AF mapping

Of the three means of signal contamination explored in this study, observed rotational activity was most degraded by far-field deflections. This is due to the ambiguity introduced when a given method attempts to mark the phase of an electrogram with multiple peaks and complex morphologies. During AF, signals have been shown to exhibit such complex morphologies as well as deflections due to far-field activity (Narayan et al., 2011). While some methods explicitly attempt to filter out these far-field effects (Gray et al., 1995), it is often difficult to distinguish far-field from near-field activity in electrograms. Innovations in accurately computing the phase of complex electrograms with significant far-field contamination could lead to more accurate phase maps, particularly in cases with sub-optimal electrode contact.

In addition, it should be possible to further investigate the effect of signal contamination and fidelity using computational studies. Such studies, successfully applied to investigate multiple aspects of activation mapping (Aslanidi et al., 2011; Gonzales et al., 2014; Labarthe et al., 2014; McDowell et al., 2015; Rappel et al., 2015; Boyle et al., 2018), can address questions not easily accessible in clinical studies, including questions regarding spatial heterogeneities, wave front collisions, and spatial resolution (Rappel and Narayan, 2013; Roney et al., 2017). We are currently planning to use computational studies to address these questions.

### Limitations

The current study has several limitations. First, it has been argued that rotational sources identified with phase maps can be artificial and due to chance (Kuklik et al., 2016). While transient rotational activity can arise in phase maps, rotational activity observed in AF in this study occurred intermittently throughout the entire 20 s duration, with conserved chirality and spatial location, which is extremely unlikely due to chance. Moreover, clinical intervention (ablation) at the rotational site terminated persistent AF, supporting a mechanistic role in perpetuating the fibrillatory state. Second, we analyzed AF from a limited number of patients since the study was designed to develop a fidelity measure to quantify which signal contaminants most affect stable rotational activity in an index case with high quality electrograms and few contaminants. While we cannot generalize from this computational study to all specific signal contaminants, our prior work in larger series shows similar characteristics at sites of termination of persistent AF (Alhusseini et al., 2017; Kowalewski et al., 2018; Zaman et al., 2018). We are planning a systematic analysis of episodes in more patients, with varying degrees of signal complexity.

## Author contributions

DV and W-JR conceived the study. SN collected the data. Model construction was performed by DV, MA, and W-JR. Data analysis was performed by DV and MA. The manuscript was written by DV, SN, and W-JR. All authors reviewed and approved the manuscript.

### Conflict of interest statement

W-JR and SN are coauthors of intellectual property owned by the University of California Regents, licensed to Abbott. W-JR and DV have filed patent applications related to the identification of fibrillation sources. SN received honoraria from Medtronic and St. Jude Medical. The reviewer MS and handling Editor declared their shared affiliation at the time of the review. The remaining author declares that the research was conducted in the absence of any commercial or financial relationships that could be construed as a potential conflict of interest.

## Supplementary material

Supplementary Video Animated phase maps of AF for 4 seconds of data. Color scales from 0 (blue) to 2π (red), with primary phase singularities marked with a green dot (CCW) or blue dot (CW) as chosen by K-means clustering. Targeted ablation at the CCW rotational pattern resulted in AF termination.
